# Using Semantic Web technology to support icd-11 textual definitions authoring

**DOI:** 10.1186/2041-1480-4-11

**Published:** 2013-04-21

**Authors:** Guoqian Jiang, Harold R Solbrig, Christopher G Chute

**Affiliations:** 1 Department of Health Sciences Research, Mayo Clinic, 200 First St SW, Rochester, MN, 55905, USA

**Keywords:** Semantic Web Technology, RDF, SPARQL, ICD-11, SNOMED CT, DBpedia

## Abstract

The beta phase of the 11^th^ revision of International Classification of Diseases (ICD-11) intends to accept public input through a distributed model of authoring. One of the core use cases is to create textual definitions for the ICD categories. The objective of the present study is to design, develop, and evaluate approaches to support ICD-11 textual definitions authoring using Semantic Web technology. We investigated a number of heterogeneous resources related to the definitions of diseases, including the linked open data (LOD) from DBpedia, the textual definitions from the Unified Medical Language System (UMLS) and the formal definitions of the Systematized Nomenclature of Medicine—Clinical Terms (SNOMED CT). We integrated them in a Semantic Web framework (i.e., the Linked Data in a Resource Description Framework [RDF] triple store), which is being proposed as a backend in a prototype platform for collaborative authoring of ICD-11 beta. We performed a preliminary evaluation on the usefulness of our approaches and discussed the potential challenges from both technical and clinical perspectives.

## Introduction

The 11^th^ revision of International Classification of Diseases (ICD-11) was officially launched by the World Health Organization (WHO) in March 2007 [1]. A 3-tiered content model (see more details in Background section) has been proposed and discussed under WHO Topic Advisory Group on Health Informatics and Modeling [2]. The purpose of the ICD-11 content model is to present the knowledge that underlies the definitions of an ICD entity. Starting in May 2012, the beta phase of the ICD-11 revision intends to accept public input through a distributed model of authoring. One of the core use cases is to create the textual definitions for each ICD category. The parameter *textual definitions* is described by WHO as, “Each ICD concept will be accompanied by a written definition of its descriptive characteristics. This full text definition enables human users to understand the meaning of a concept for classification, translation and other reasons [2].”

The provision of textual definitions has been regarded as one of important criteria for measuring the quality of a terminology/ontology [3]. A well-structured human-readable definition, by distinguishing one entity from another, may serve as the basis for the formal definition (i.e., a computational definition of a class or category, usually expressed in description logic) of an entity. While human-readable definitions may be more complete and detailed than formal definitions, there still should not be any discordance between them.

The objective of the present study is to design, develop, and evaluate approaches to support ICD-11 textual definitions authoring using Semantic Web technology. We investigate a number of heterogeneous resources related to the definitions of diseases, including the linked open data (LOD) from DBpedia, the textual definitions from the Unified Medical Language System (UMLS) and the formal definitions of the Systematized Nomenclature of Medicine—Clinical Terms (SNOMED CT). We integrate them in a Semantic Web framework (i.e., the Linked Data in a Resource Description Framework [RDF] triple store), which is being proposed as a backend in a prototype platform for collaborative authoring of ICD-11 beta. We perform a preliminary evaluation on the usefulness of our approaches and discuss the potential challenges from both technical and clinical perspectives.

## Background

### ICD-11 and its content model

Historically, ICD was developed to support international comparison of mortality statistics. WHO has embraced a broadened set of use cases to drive ICD-11 development, including scientific consensus of clinical phenotype (definition and criteria), public health surveillance (e.g., mortality and morbidity), and clinical data aggregation [4].

Each ICD entity can be seen from different dimensions. The content model represents each one of these dimensions as a parameter. Currently, there are 13 defined main parameters in the content model to describe a category in ICD. Table 1 illustrates that “Textual Definitions” is one of main parameters for describing an ICD category.

**Table 1:** The ICD-11 content model main parameters

1	ICD Entity Title
2	Classification Properties
3	Textual Definitions
4	Terms
5	Body System/Structure Description
6	Temporal Properties
7	Severity of Subtype Properties
8	Manifestation Properties
9	Causal Properties
10	Functioning Properties
11	Specific Condition Properties
12	Treatment Properties
13	Diagnostic Criteria

### SNOMED CT and its canonical forms

SNOMED CT is the most comprehensive, clinically oriented medical terminology system. It is owned and maintained by the International Health Terminology Standard Development Organization (IHTSDO) [5], and is now specified in the US, UK, and several other countries as a preferred or required terminology for coding clinical problems and other aspects of the electronic health record. IHTSDO and WHO signed a collaborative agreement in July 2010, aimed at enabling harmonization of WHO Classifications and SNOMED CT, which essentially establishes SNOMED CT as the core of the ontological component of ICD [6].

SNOMED CT adopted a description logic foundation that has allowed its curators to formally represent concept meanings and relationships. SNOMED CT proposed the canonical (or normal) forms for its concept codes [7]. A normal form is a view that can be generated by maximally decomposing any valid expression by applying a set of logical transformation rules. The purpose of generating normal forms is to facilitate complete and accurate retrieval of precoordinated and postcoordinated SNOMED CT expressions from clinical records or other resources. Two alternative normal forms are proposed: the long canonical form and the short canonical form. We used the short canonical form to generate the structured definition for a SNOMED CT code (see the Methods section).

### iCAT and ICD-11 alpha authoring

WHO initially adopted Web-Protégé for the alpha phase of ICD-11 development and the tool is called “iCAT”. iCAT is a variant of Web-Protégé, which is a web-based application using Google Web Toolkit (GWT) technology [8].

For the alpha process, the user community is relatively small as the main task is to augment rubric definitions and review of elements in the foundation component of the ICD. However, in the beta phase, the ICD will be reviewed publicly and the number of user community could potentially be large. The scalability issue of the iCAT tool will be challenged, as multiple users work on the same copy of an evolving ICD category in that tool.

### LexWiki and the proposal-based mechanism

LexWiki is an effort led by Mayo Clinic for development of a collaborative authoring platform for large-scale biomedical terminologies [9]. The LexWiki environment based on Semantic MediaWiki [10] enables the wider community to make both structured and unstructured proposals on the definitions of classes and property values, suggest new values, and make corrections to the current ones. LexWiki currently is at the core of community-based development of Biomedical Grid Terminology [11] and has also been successfully implemented to support the Common Terminology Criteria for Adverse Events revision project [12] and the Clinical Data Interchange Standards Consortium (CDISC) Shared Health and Research Electronic Library project [13].

### Semantic Web technology

The World Wide Web Consortium (W3C) is the main international standards organization for the World Wide Web. Its goal is to develop interoperable technologies and tools as well as specifications and guidelines to lead the Web to its full potential. W3C recommendations have several maturity levels: Working Draft, Candidate Recommendation, Proposed Recommendation, and W3C Recommendation. RDF, a W3C recommendation, is a directed, labeled graph data format for representing information in the Web [14]. The Linked Data uses the RDF data model that encodes data in the form of subject, predicate, and object triples. SPARQL (SPARQL Protocol and RDF Query Language) is a query language for RDF graphs. SPARQL queries are expressed as constraints on graphs, and return RDF graphs or sets as results. SPARQL 1.0 has been a W3C recommendation whereas SPARQL 1.1 is a Working Draft [15,16]. Triplestore is a database for the storage and retrieval of RDF metadata, ideally through standard SPARQL query language.

## Methods

### Proposed collaborative authoring framework for ICD-11 Beta

**Figure 1:**
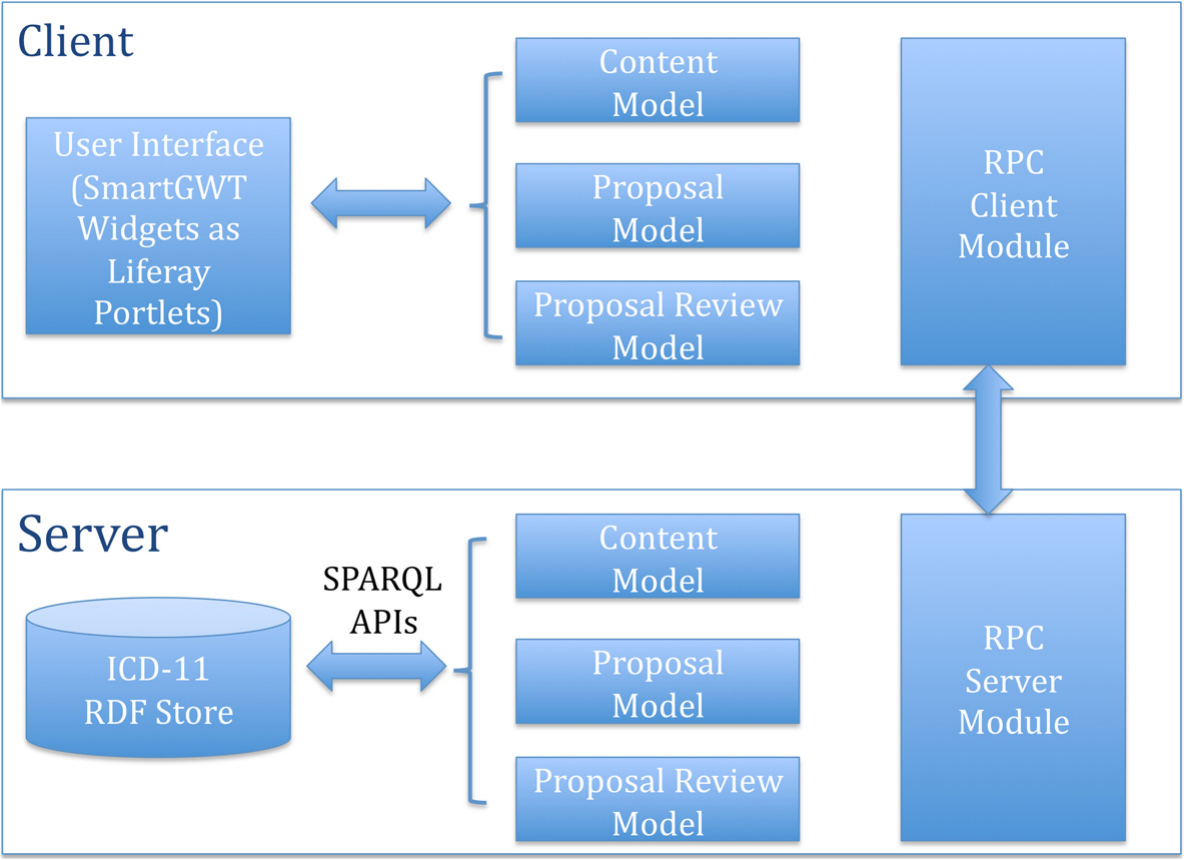
System architecture of proposed ICD-11 beta collaborative authoring platform.

Figure 1 shows the system architecture we proposed for the ICD-11 beta collaborative authoring platform. In the client side, we chose to use the SmartGWT rich widget library [17] and Liferay portal system [18] to develop the user interface. In the server side, we chose to use an RDF store for ICD-11 contents and metadata persistence. We used the GWT Remote Procedure Calls technology to realize the communication between the client and the server. Besides utilizing the ICD-11 content model, we enable a proposal provenance model. The model is used to represent the provenance data required for the implementation of a proposal-based authoring mechanism informed by our previous work on the LexWiki system [9].

As the ICD-11 beta will be based upon the contents of the ICD-11 alpha, we developed a transformation pipeline to convert the ICD-11 alpha data into the Semantic Web format. The ICD-11 alpha data is rendered in the MySQL relational database format and the db dump is available for download [19]. We utilized the D2R technology [20], defined a D2RQ mapping file, and converted the relational database to the RDF triples. Since the D2R server did not support those SPARQL 1.1 features required for the authoring purpose (i.e., the RDF graph update operations), we dumped the RDF triples utilizing the RDF dump feature of the D2R.

In a prototype implementation, we adopted the 4store that is a scalable open source RDF database developed at the Garlik [21]. We loaded the RDF dump from the D2RQ transformation using the 4store built-in import script. With the ICD-11 contents loaded in the RDF store, we were able to define the standard SPARQL queries to access the contents through the 4store built-in SPARQL endpoint, and to utilize its SPARQL 1.1 features for the authoring purpose. Table 2 shows a SPARQL query example to get all chapter labels and codes of ICD-11. The similar queries are used to extract the data to build the ICD-11 category hierarchy in the user interface.

**Table 2:** The SPARQL query example to get all chapter labels and codes of ICD-11

**SPARQL Query**	**Note**
SELECT DISTINCT ?label ?code	To get all chapter labels and codes of ICD-11
{ GRAPH <http://who.int/icd>	
{ <http://who.int/icd#ICDCategory>	
<http://who.int/icd/vocab/resource/DIRECT-SUBCLASSES> ?child.	
?child <http://who.int/icd/vocab/resource/DIRECT-SUPERCLASSES> ?parent.	
?child rdfs:label ?label .	
?child <http://who.int/icd#icdCode> ?code.	
} } ORDER BY ?label	

### Proposed system design for textual definitions authoring

**Figure 2:**
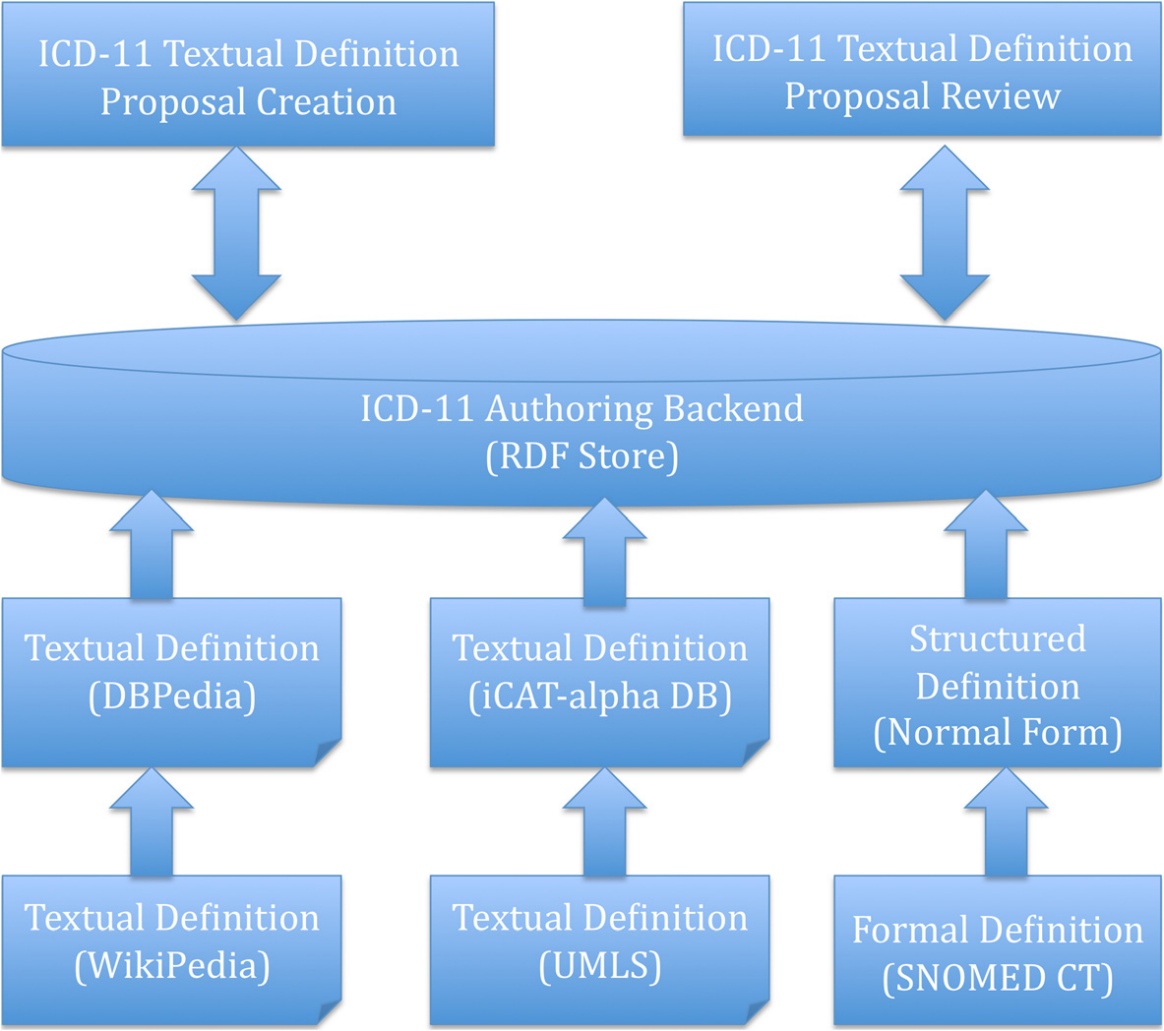
System design for the use case of textual definitions authoring.

Figure 2 shows the system design for the use case of textual definitions authoring, chosen as an initial prototype. We integrated 3 heterogeneous resources related to the definitions of diseases, including the LOD from DBpedia [22], the textual definitions from the UMLS [23], and the formal definitions of SNOMED CT [5].

#### Textual definitions from DBpedia

To utilize the LOD data in DBpedia, we accessed its SPARQL endpoint at http://dbpedia.org/sparql. We defined a SPARQL query and extracted those instances with the type of disease. Table 3 shows the SPARQL query that retrieves the information of label, abstract, MeSH ID, and the corresponding WikiPedia link for the instances with the type of disease (defined in the DBpedia ontology as http://dbpedia.org/ontology/Disease). We consider the abstract information closely corresponds to the definition. We used the MeSH ID as an anchor to map the DBPedia disease definitions to those corresponding codes in both SNOMED CT and ICD-10 through the UMLS concept unique identifiers (CUIs).

**Table 3:** A SPARQL query against the SPARQL endpoint of the DBpedia to extract the disease definition information in the language of English

**SPARQL Query**	**Note**
SELECT DISTINCT ?label ?abstract ?meshId ?wikipediaLink	To extract the disease definition information in the language of English from Dbpedia
WHERE {	
?s a <http://dbpedia.org/ontology/Disease>.	
?s rdfs:label ?label.	
?s <http://dbpedia.org/ontology/abstract> ?abstract.	
?s <http://dbpedia.org/ontology/meshId> ?meshId .	
?wikipediaLink <http://xmlns:m.com/foaf/0.1/primaryTopic> ?s.	
FILTER (langMatches(lang(?label), "en") && langMatches(lang(?abstract), "en"))	
}	

#### Textual definitions from UMLS

The textual definitions from UMLS had already been imported into the original ICD-11 alpha database for a portion of ICD categories. Therefore, we were able to access the textual definitions just by defining the SPARQL queries against the RDF dump that was loaded into the ICD-11 RDF store as described in above section. Table 4 shows a SPARQL query example that extracts the definition and its metadata for a specific ICD category “A19 – Miliary tuberculosis”. Table 5 shows the query results.

**Table 4:** A SPARQL query example to extract the definition and its metadata for a specific ICD category, A19 – Miliary tuberculosis

**SPARQL Query**	**Note**
SELECT DISTINCT ?label ?definitionContent ?ontologyId ?termId	To extract the definition and its metadata for a specific ICD category, A19 – Miliary tuberculosis
{ GRAPH <http://who.int/icd>	
{ <http://who.int/icd#A19> <http://who.int/icd#definitionPrefilled> ?prefilledDefinition .	
<http://who.int/icd#A19> rdfs:label ?label .	
?prefilledDefinition <http://who.int/icd#label> ?definitionContent;	
<http://who.int/icd#ontologyId> ?ontologyId;	
<http://who.int/icd#termId> ?termId;	

}}	

**Table 5:** The query results for the definition of the ICD category A19 – Miliary tuberculosis

**Label**	**DefinitionContent**	**OntologId**	**TermId**
A19. Miliary tuberculosis	An acute form of TUBERCULOSIS in which minute tubercles are formed in a number of organs of the body due to dissemination of the bacilli through the blood stream.	UMLS/MSH2008_2008_02_04	C0041321

#### Structured definitions from the SNOMED CT

We utilized the data files and the canonical table file of the 20100731 International Release of SNOMED CT. We defined simple grammatical rules that can be used to render those elements in the short canonical form into the structured definition that is more human-readable to the domain professionals. Table 6 shows the structured definition of “Acute myocardial infarction” derived from its short canonical form.

**Table 6:** The structured definition of “Acute myocardial infarction” derived from its short canonical form

**Definition**	**Note**
Acute myocardial infarction	The structured definition of “Acute myocardial infarction” derived from its short canonical form
is a Disease	
that has Clinical course of Sudden onset AND/OR short duration	
that has Associated morphology of Acute infarct	
and has Finding site of Myocardium structure	

We mapped the SNOMED CT codes and their corresponding structured definitions with the ICD categories represented by the ICD-10 codes through using the UMLS CUIs. We then rendered the mappings and definitions into the RDF triples and loaded them into the ICD-11 RDF store in a separate graph model using the 4store built-in import script. Table 7 shows the mapping between the ICD category “I21” and the SNOMED CT code “57054005” and its structured definition rendered in the RDF triples.

**Table 7:** The RDF triples in Turtle format rendered for the mapping between the ICD category “I21” and the SNOMED CT code “57054005”and its structured definition

**RDF Triples**	**Note**
<http://who.int/icd#I21> <http://who.int/icd#icdCode> "I21";	The RDF triples in Turtle format rendered for the mapping between the ICD category “I21” and the SNOMED CT code “57054005”and its structured definition
<http://who.int/icd#definitionPrefilled> _:b0672.	
_:b0672 <http://who.int/icd#label>	
"Acute myocardial infarction Is a Disease and	
has Clinical course of Sudden onset AND/OR short duration	
that has Associated morphology of Acute infarct	
and has Finding site of Myocardium structure " ;	
<http://who.int/icd#ontologyId> "SNOMED CT" ;	
<http://who.int/icd#termId> "C0155626";	
<http://who.int/icd#sctId> "57054005".	

Note that the label text is wrapped for the display purpose.

### System evaluation

We performed a preliminary evaluation on the usefulness of our approaches on textual definitions authoring in the following aspects. First, we evaluated the coverage of each definition resource. Second, we performed a case study on 2 example ICD categories. We linked the definitions extracted from all 3 resources with each of the 2 categories and profiled the definitions using the ICD-11 content model. The purpose of this evaluation is to illustrate the potential gap between the textual definitions and the formal definitions.

## Results

**Figure 3:**
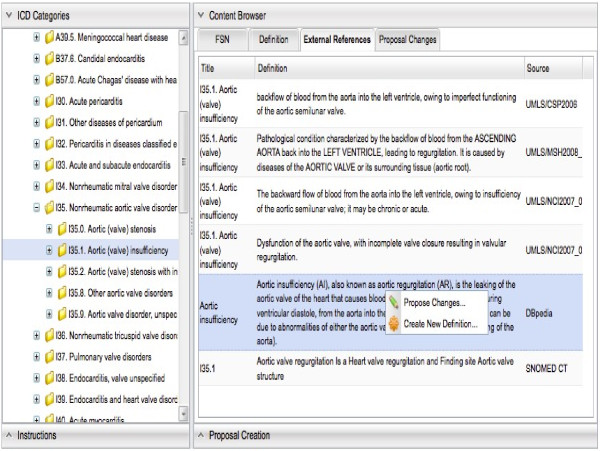
A screenshot of an initial user interface prototype illustrating how the textual definitions are leveraged in our proposed collaborative authoring system for the ICD-11 beta project.

We successfully transformed the ICD-11 contents into the Linked Data in a RDF store, which is utilized as the backend in a prototype of our proposed collaborative authoring system for ICD-11 beta project. To support the use case of textual definitions authoring, we developed the approaches that integrated 3 resources using Semantic Web technology. The resources comprised the disease definitions from the LOD data in the DBpedia, the textual definitions from the UMLS and the structured definitions from the SNOMED CT. Figure 3 shows a screenshot of an initial user interface prototype illustrating how the textual definitions are leveraged in our proposed collaborative authoring system.

From the LOD data in DBpedia, we extracted 2,735 distinct disease definitions and labels in the language of English, as well as their corresponding MeSH Ids and Wikipedia links. Using the MeSH IDs, we were able to link the textual definition from the DBpedia with ICD categories through the UMLS CUIs. In total, the disease labels and definitions correspond to 2,463 distinct MeSH IDs, which were mapped to 1,069 ICD categories represented by the ICD-10 codes.

From the ICD-11 RDF store, we identified 1,487 textual definitions for 1,278 distinct ICD categories. The textual definitions were mainly from 7 different coding schemes of the UMLS, including the NCI Thesaurus (UMLS/NCI2007_05E), the MeSH (UMLS/MSH2008_2008_02_04), the Gene Ontology (UMLS/GO2007_02_01), and the Computer Retrieval of Information on Scientific Projects (UMLS/CSP2006), etc.

From the canonical table of the SNOMED CT, it contained the short canonical forms of 96,235 SNOMED CT concept ids from the branch of “Clinical Finding”. Utilizing the grammatical rules we defined, we were able to transform the short canonical forms into the structured definition for each of the codes. Through the UMLS CUIs, we mapped 5,778 ICD categories represented by the ICD-10 codes to 6,122 SNOMED CT concept ids.

As a case study, we randomly selected 2 ICD categories that had the definitions from all 3 sources, the “I35.0 Aortic (valve) stenosis” and the “N17-N19 Renal failure”. Each category had 5 definition entries. We profiled each entry of the definitions using the ICD-11 content model parameters. Table 8 and Table 9 show the profiling results. The results indicated that the textual definitions were more detailed than the structured definitions derived from the formal definitions. In addition, we found that most of definitions specified the supertypes but the supertypes varied in different granularity. Taking the example from Table 8, the supertypes specified for the “Aortic valve stenosis” include “a valvular heart disease”, “a pathological constriction” or “a disease”.

**Table 8:** The linked definition resources to the ICD category “I35.0 Aortic (valve) stenosis”

**Source**	**Definition type**	**Definition**	**ICD-11 parameters involved**
DBpedia	Textual definition	Aortic valve stenosis (AS) is a type of valvular heart disease characterized by an abnormal narrowing of the aortic valve opening.	Supertype, Morphology, anatomical site
UMLS/MSH2008_2008_02_04	Textual definition	A pathological constriction that can occur above (supravalvular stenosis), below (subvalvular stenosis), or at the AORTIC VALVE. It is characterized by restricted outflow from the LEFT VENTRICLE into the AORTA.	Supertype, Morphology, Anatomical site
UMLS/NCI2007_05E	Textual definition	Narrowing of the orifice of the aortic valve or of the supravalvular or subvalvular regions.	Morphology, Anatomical site
UMLS/CSP2006	Textual definition	Constriction in the opening of the aortic valve or of the supravalvular or subvalvular regions.	Morphology, Anatomical site
SNOMED CT	Structured definition	Aortic valve stenosis is a Disease that has Associated morphology of Stenosis and has Finding site of Aortic valve structure.	Supertype, Morphology, Anatomical site

**Table 9:** The linked definition resources to the ICD category N17-N19 Rental failure

**Source**	**Definition type**	**Definition**	**ICD-11 parameters involved**
DBpedia	Textual definition	Renal failure or kidney failure (formerly called renal insufficiency) describes a medical condition in which the kidneys fail to adequately filter toxins and waste products from the blood. The two forms are acute and chronic; a number of other diseases or health problems may cause either form of renal failure to occur. Renal failure is described as a decrease in the glomerular filtration rate. Biochemically, renal failure is typically detected by an elevated serum creatinine level. Problems frequently encountered in kidney malfunction include abnormal fluid levels in the body, deranged acid levels, abnormal levels of potassium, calcium, phosphate, and (in the longer term) anemia. Depending on the cause, hematuria (blood loss in the urine) and proteinuria (protein loss in the urine) may occur. Long-term kidney problems have significant repercussions on other diseases, such as cardiovascular disease.	Supertype, anatomical site, causal, manifestation, diagnostic criteria
UMLS/MSH2008_2008_02_04	Textual definition	A severe irreversible decline in the ability of kidneys to remove wastes, concentrate URINE, and maintain ELECTROLYTE BALANCE; BLOOD PRESSURE; and CALCIUM metabolism. Renal failure, either acute (KIDNEY FAILURE, ACUTE) or chronic (KIDNEY FAILURE, CHRONIC), requires HEMODIALYSIS.	Supertype, anatomical site, severity, temporal, treatment
UMLS/NCI2007_05E	Textual definition	Acute or chronic condition, characterized by the inability of the kidneys to adequately filter the blood substances, resulting in uremia and electrolyte imbalances. Acute renal failure is usually associated with oliguria or anuria, hyperkalemia, and pulmonary edema. Chronic renal failure is irreversible and requires hemodialysis.--2004	Supertype, anatomical site, manifestation, temporal, treatment
UMLS/CSP2006	Textual definition	Inability of a kidney to excrete metabolites at normal plasma levels under conditions of normal loading or inability to retain electrolytes under conditions of normal intake.	Anatomical site, diagnostic criteria
SNOMED CT	Structured definition	Renal failure syndrome is a Renal impairment.	Supertype

## Discussion

In this study, we demonstrated that how Semantic Web technology was leveraged to integrate heterogeneous disease definition data to support ICD-11 textual definitions authoring. With the capacity of the RDF store, we were able to integrate multiple, heterogeneous disease definition resources in an agile manner. The underlying RDF model encoding of knowledge in the form of triples plays a key role on this as the RDF can be used as a schema-less data representation format. This ensures the flexibility of our system. Using the powerful SPARQL query language, we were able to access the definition elements in the ICD-11 RDF store, as well as the external LOD data services.

The textual definitions extracted from DBpedia are a typical example of traditional human readable definitions generated using a crowdsourcing model. The definitions are actually harvested by DBpedia from Wikipedia, one of the largest collaborative authoring platforms in the world. DBpedia is a Linked Data project aiming to extract structured contents from the information created as part of the Wikipedia project. DBpedia allow users to query relationships and properties associated with Wikipedia resources, including links to other related datasets [24].

Using the LOD service of DBpedia, we can easily extract the shared definition data through standard SPARQL queries for the purpose of the ICD-11 use case. We found that the type “Disease” and the predicate “meshId” defined in DBpedia ontology are very useful for the extraction process. The MeSH IDs provided a mapping bridge between the coding schemes like SNOMED CT and ICD, which are utilized in this project.

In addition, the multilingual definitions are available for most of disease instances in DBpedia, though we just extracted those in the language of English. For example, the definitions of the “Aortic valve stenosis” were available in 12 languages in DBpedia. We consider this may provide added values for the ICD-11 project, as the multilingual support is one of critical requirements for the ICD-11 content authoring.

We also argue that the ICD-11 project may potentially take advantage of the crowdsourcing model of Wikipedia. Using this model, each ICD-11 category would be seeded as a Wikipedia page for public input and the definitions of the categories would be harvested using the DBpedia. And then the WHO Topic Advisory Groups may just play a role in reviewing the harvested definitions to ensure the quality of the data.

The textual definitions from the UMLS had been extracted using the mappings between the ICD-10 and other coding schemes in the UMLS through their shared CUIs. As the example illustrated in above section, an ICD code can have multiple definitions from multiple coding schemes identified. We consider this an important source of definitions as the references for the ICD-11 use case though basically the definitions may have been authored in different contexts for the different purpose.

We developed an approach to generate the structured definitions from the formal definitions of SNOMED CT concept codes. The task of generating texts from ontologies has been called *ontology verbalization*. A notable application of ontology verbalization has been controlled natural languages (CNL) as a means of both reading and authoring ontologies. For instance, Attempto Controlled English [25] is a typical example of such a CNL application. In biomedical domain, Stevens et al. developed an approach to take the logical description of entities in an OWL (Web Ontology Language) –based ontology and automatically generate text-based definitions in fluent natural language [26]. In this study, we chose to use the short canonical forms from the original distribution of SNOMED CT for the structured definition generation. As the short canonical form reduces complexity and duplication in the defining characteristics without losing any of the information embedded in the definition, we consider that the structured definition derived from the canonical form would represent well the core meaning of the corresponding concept code.

In addition, SNOMED CT concept codes have been used as the ontological component of ICD-11 to provide references to formal definition of terms and relationships for ICD-11 categories. In a previous study, we performed a case study on ICD-11 anatomy value set extraction from SNOMED CT [27]. Theoretically, each ICD-11 category would have a corresponding mapping to a SNOMED CT code. In this context, we consider our structured definition generation approach will be helpful to facilitate the mapping process by providing human readable definitions.

In summary, Semantic Web technology provides a scalable framework to allow the successful integration of the heterogeneous definitions resources in support of collaborative authoring of ICD-11 textual definitions. Our next steps in the future will focus on 1) having a further evaluation of the crowdsourcing model of Wikipedia/DBpedia for ICD-11 textual definitions from public input; 2) having a more rigorous evaluation of the quality and usefulness of the definition resources; 3) designing and developing a user interface that allows the ICD community to leverage the definition resources to produce the solid textual definitions for the ICD-11 categories; 4) developing methods and tools to allow the system to check the consistency between the ICD-11 textual definitions and their formal definitions; and 5) developing the methods and tools to support the mapping between the ICD-11 categories and the SNOMED CT codes leveraging both textual and formal definitions.

## Competing interests

The authors declare that they have no conflict of interest.

## Authors’ contributions

GJ and HRS conceived the study, performed data analysis and drafted manuscript; CGC provided institutional support and reviewed the manuscript. All authors have read and approved the final manuscript.
